# A Point-of-Care Testing Device Utilizing Graphene-Enhanced Fiber Optic SPR Sensor for Real-Time Detection of Infectious Pathogens

**DOI:** 10.3390/bios13121029

**Published:** 2023-12-14

**Authors:** Shiyu Jiang, Siyu Qian, Shunning Zhu, Jinxin Lu, Yunxin Hu, Cheng Zhang, Yikai Geng, Xuefeng Chen, Ying Guo, Zhaoliang Chen, Jie Pu, Zhendong Guo, Shengchun Liu

**Affiliations:** 1 Heilongjiang Provincial Key Laboratory of Metamaterials Physics and Device, Heilongjiang University, Harbin 150080, China; jiangshiyv@163.com (S.J.); a1020318309@gmail.com (S.Z.); 18845594580@163.com (J.L.); huyunxinmail@163.com (Y.H.); 18032866837@163.com (Y.G.); chenxuefeng_vip@163.com (X.C.); guoying@hlju.edu.cn (Y.G.); 2 School of Electronic Engineering, Heilongjiang University, Harbin 150080, China; 3 Changchun Veterinary Research Institute, Chinese Academy of Agricultural Sciences, Changchun 130122, China; zc1349@foxmail.com (C.Z.); zl981429835@163.com (Z.C.); pujie17765367564@163.com (J.P.); guozd@foxmail.com (Z.G.)

**Keywords:** surface plasmon resonance, infectious pathogen detection, portable device, graphene film, point-of-care testing

## Abstract

Timely detection of highly infectious pathogens is essential for preventing and controlling public health risks. However, most traditional testing instruments require multiple tedious steps and ultimately testing in hospitals and third-party laboratories. The sample transfer process significantly prolongs the time to obtain test results. To tackle this aspect, a portable fiber optic surface plasmon resonance (FO-SPR) device was developed for the real-time detection of infectious pathogens. The portable device innovatively integrated a compact FO-SPR sensing component, a signal acquisition and processing system, and an embedded power supply unit. A gold-plated fiber is used as the FO-SPR sensing probe. Compared with traditional SPR sensing systems, the device is smaller size, lighter weight, and higher convenience. To enhance the detection capacity of pathogens, a monolayer graphene was coated on the sensing region of the FO-SPR sensing probe. Severe acute respiratory syndrome coronavirus 2 (SARS-CoV-2) was used to evaluate the performance of the portable device. The device can accurately detect the SARS-CoV-2 spike S1 protein in phosphate-buffered saline (PBS) and artificial saliva within just 20 min, and the device successfully detected cultured SARS-CoV-2 virus. Furthermore, the FO-SPR probe has long-term stability, remaining stable for up to 8 days. It could distinguish between the SARS-CoV-2 spike protein and the MERS-CoV spike protein. Hence, this FO-SPR device provides reliable, rapid, and portable access to test results. It provides a promising point-of-care testing (POCT) tool for on-site screening of infectious pathogens.

## 1. Introduction

Potential pandemic pathogens, such as human influenza A (H5N1) [1], Middle East respiratory syndrome (MERS) [2], severe acute respiratory syndrome (SARS) [3], and severe acute respiratory syndrome coronavirus 2 (SARS-CoV-2) [4] have continued to threaten human health and became the major public health concerns [5]. However, current conventional pathogen detection devices, including cell cultivation, enzyme-linked immunosorbent assay (ELISA), and reverse transcription-polymerase chain reaction (RT-PCR), are usually performed in medical laboratories. They not only need bulky testing equipment but also need professional experimental technologists. These methods suffer from some drawbacks such as long turnaround time, high cost, and complex operations [6]. These limitations make laboratory-based detection nearly impossible for timely on-site inspection [7]. Therefore, developing a portable and user-friendly device for rapid and real-time field detection is necessary.

Point-of-care testing (POCT) is a decentralized detection form of modern laboratory medicine that can be applied directly at the patient’s bedside or the screening site [8]. It offers the advantages of rapid acquisition and cost-effectiveness [9]. As a result, rapid and portable diagnostic POCT analysis methods such as test strips are widely used in medicine clinics [10,11,12]. However, the ability to provide only “yes/no” test results seriously restricts further application in clinical medical diagnosis and quantitative analysis.

As an effective method for label-free real-time medical diagnostics, surface plasmon resonance (SPR) biosensors have proven themselves to be a powerful tool for quantitative pathogen detection [13,14,15]. Shahriar Mostufa et al. reported a hybrid TiO_2_/Au/graphene layer-based SPR sensor with improved sensitivity and capability for cancer detection is presented [16]. Haneul Yoo et al. reported a reusable sensor chip using a ferromagnetic pattern to capture a layer of magnetic particles. The proposed SPR substrate successfully detects the H1N1 influenza virus [17]. Recently, Awadhesh Kumar et al. proposed a silicon nitride-BP-based SPR biosensor for highly sensitive virus SARS-CoV-2 detection [18]. Commercial SPR instruments exhibit topnotch sensitivity capabilities in pathogen detection. Nevertheless, they are usually bulky, optically designed, and mechanically complex, making them unsuitable for portable detection [19]. Fortunately, fiber optic surface plasmon resonance (FO-SPR) sensing has the advantage of smaller size, lower costs, and simpler optical designs [20,21]. Hence, FO-SPR sensors are promising portable tools for the detection of infectious pathogens, which can easily be adapted to POCT [22,23,24]. In order to further improve the sensitivity of FO-SPR detection to meet the requirements of POCT, the sensing region was coated with monolayer graphene. The graphene’s large surface-to-volume ratio remarkably increases the absorption of biomolecules on the sensor [25,26]. Moreover, graphene is biocompatible [27], enabling bio-functionalization. How to use the FO-SPR sensor to build a portable high-sensitivity pathogen POCT device is a more anticipated research issue for scientists.

In this paper, a portable FO-SPR POCT device was developed for real-time detection of highly infectious pathogens. The device integrated the following parts: (I) FO-SPR sensing component; (II) signal acquisition and processing system; (III) embedded power supply unit. The sensing signals are processed and analyzed by a program written on the LabVIEW-based platform to support automated data acquisition and real-time visualization. Since each part requires different power, a variable voltage circuit is used to distribute the supply voltage for the device rationally. FO-SPR sensing probes are used for infectious pathogen detection. In an effort to further improve the sensitivity, the monolayer graphene is coated on the fiber sensing probe. Meanwhile, the selectivity is achieved by immobilizing specific antibodies onto the sensing probe through 1-pyrenebutyric acid N-hydroxysuccinimide ester (PBASE). The integrated FO-SPR device manifests enormous potential in the POCT of highly infectious pathogens.

## 2. Materials and Methods

### 2.1. Materials and Reagents

Cu-based monolayer graphene (Precoating PMMA) was bought from XFNANO Materials Tech Co., Ltd. (Nanjing, China). 1-Pyrenebutyric acid N-hydroxysuccinimide ester (PBASE) was bought from Alfa Aesar (Shanghai, China). Methanol, Isopropyl alcohol, Ferric chloride (FeCl_3_⸱6H_2_O), Phosphate-buffered saline (PBS), 11-Mercaptoundecanoic acid (11-MUA), N-hydroxysulfosuccinimide (NHS), 1-(3-dimethylaminopropyl)-3-ethylcarbodiimide hydrochloride (EDC), and bovine serum albumin (BSA) was purchased from Aladdin (Shanghai, China). Artificial saliva was purchased from An Yongbo Technology Co., Ltd. (Xiamen, China). SARS-CoV-2 spike S1 antibody (Cat: 40150-R007), SARS-CoV-2 Spike S1 Protein (Cat: 40591-V08H), and MERS-CoV Spike S1 Protein (Cat: 40069-V08H) were obtained from Sino Biological Inc. (Beijing, China). Deionized water was obtained by using the ultrapure water production system and autoclaved at 121 °C for 20 min. Other reagents purchased in this study were analytical pure standards if not otherwise specified.

### 2.2. SARS-CoV-2 Culture and Inactivation

The SARS-CoV-2 virus (BetaCoV/Beijing/IME-BJ05-2020, inactivated by heating at 56 °C for 1 h [28]) was obtained from Changchun Veterinary Research Institute, Chinese Academy of Agricultural Sciences. The inactivated viruses were stored at −80 °C before use.

### 2.3. Graphene-Based FO-SPR Sensing Probe Fabrication

The FO-SPR sensing probe used multimode fiber (YOFC Co., Ltd., Shanghai, China) with a core diameter of 400 μm and a numerical aperture of 0.37. As previously reported [29], near the end face of the FO-SPR probe, 1 cm of coating and cladding was peeled off as the sensing area. Then, the sensing area was coated with about 50 nm gold using magnetron sputtering (Quorum Q150RSPlus, East Sussex, UK) [30]. To ensure the uniformity of the coating, a unique rotating structure was made to achieve a 360° rotation of the fiber during the sputtering process. To obtain a terminal reflective sensor, a silver layer was formed on the fiber end face using magnetron sputtering technology. The silver layer was protected by encapsulation with epoxy AB adhesive, which can prevent the oxidation of the silver layer. 

The graphene film was coated on the sensing region of FO-SPR probe by the wet transfer method (as shown in Figure S1) [31]. First, the PMMA/graphene/Cu film (0.5 × 1 cm) was etched in 1 M FeCl_3_ copper etchant for 1 h to etch off the Cu foil. Then, the suspended transparent PMMA/graphene film was washed three times in a deionized water bath for at least 10 min each time to ensure the residual copper etchant was removed. Next, the PMMA/graphene film suspended in deionized water was transferred to the surface of the sensing zone of the probe and dried at room temperature. Next, the probe was heated at 145 °C for 20 min to enhance the adhesion further. Afterward, the PMMA/graphene-coated probe was immersed in acetone overnight to remove the PMMA layer. The graphene-coated probes were cleaned with acetone and isopropyl alcohol and dried under N_2_ airflow.

### 2.4. SARS-CoV-2 Antibody Bio-Functionalization on the FO-SPR Sensing Probes

**Figure 1:**
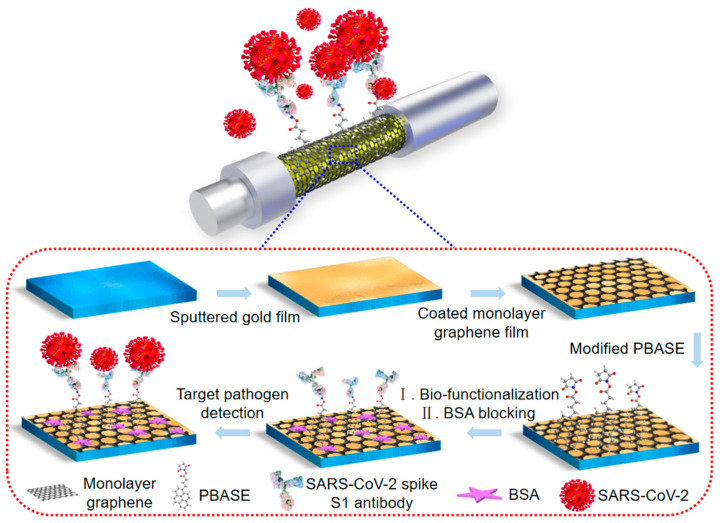
Scheme of the bio-functionalized FO-SPR sensing probe.

Figure 1 shows the bio-functionalized process of the monolayer graphene-coated FO-SPR sensing probe. FO-SPR were immersed in 2 mM PBASE in methanol for 1 h at room temperature, and then rinsed using deionized water and drying under N_2_ airflow. Then, the sensing probe was immersed in 250 μg/mL SARS-CoV-2 spike S1 antibody in PBS (pH 7.4) for 6 h and then rinsed using deionized water. To reduce the non-specific adsorption, the probes were blocked with 1 mg/mL BSA for 1 h. Finally, the bio-functionalized FO-SPR sensing probe was stored in PBS at 4 °C before use.

In order to demonstrate the higher sensitivity of graphene-coated sensing probes, the traditional 11-MUA-modified sensing probes were used as the control group (refer to Supplementary Materials). The bio-functionalized process is shown in Figure S2. The SARS-CoV-2 spike S1 antibody was immobilized on the sensing probe by 11-MUA to detect the target pathogen.

### 2.5. Portable FO-SPR Device Development

**Figure 2:**
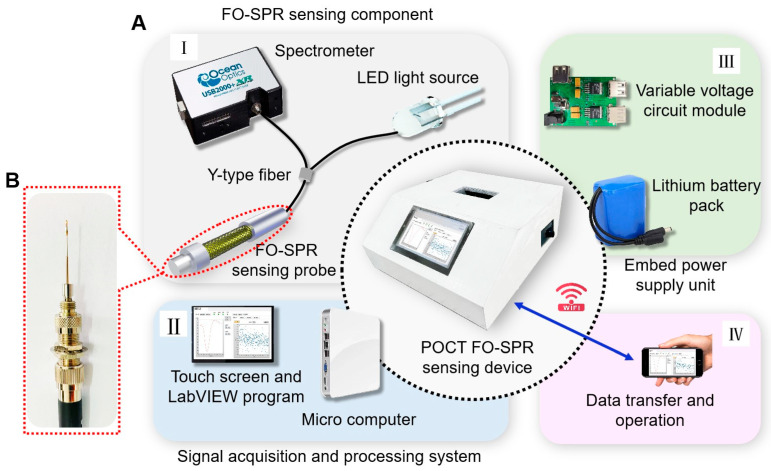
Schematic depiction of the POCT FO-SPR device. (**A**) Portable FO-SPR optical sensing scheme (**I**) FO-SPR sensing component. (**II**) Signal acquisition and processing system. (**III**) Embedded power supply unit. (**IV**) WIFI remote control module. (**B**) FO-SPR sensing probe.

The portable FO-SPR device (250 × 250 × 110 mm) is shown in Figure 2A. Namely, part I (FO-SPR sensing component) consists of a small high-power white LED light source, a miniature spectrometer (Ocean Optic USB2000+, 200–1100 nm), and a bio-functionalized FO-SPR sensing probe (Figure 2B). The sensing probe was connected to the light source and spectrometer via a fiber Y-jumper. For integrated applications, the light source and spectrometer were controlled by the signal acquisition and processing system via USB communication protocol. Part II (signal acquisition and processing system) utilized the self-made LabVIEW program to process and analyze sensor signals from the spectrometer. The program was installed on a microcomputer. The real-time detection data was displayed on a built-in touch screen through the program’s user interface, allowing the user to visualize and interpret the results. Moreover, the program controls the current and thus regulates the optical power of the light source. Part III (embedded power supply unit) was used to power the FO-SPR device. To enable outdoor on-site detection, a lithium battery pack with a capacity of 6000 mAh was used for self-powering. The variable voltage circuit was designed to distribute the power supply voltage rationally. Specifically, the lithium battery pack was connected to the input DC 5 V interface of the variable voltage circuit, and it provides three USB ports for outputting voltages of 5 V, 12 V, and 12 V. The 5 V and 12 V ports were connected to the LED light source and the microcomputer for power supply, respectively, and the other 12 V port was linked to the cooling fan. In addition, smartphones via WIFI were used to remotely control the self-made program’s operation and transfer test data, as shown in part IV. The device enclosure was designed with a 3D CAD design software (SolidWorks 2021) to mount all functional modules and reduce optical noise from the background light.

## 3. Results and Discussion

### 3.1. Pathogens Detection and Data Analysis

The portable FO-SPR device provided a user-friendly and convenient way to detect the virus in a timely manner. The FO-SPR sensing probe was designed for easy installation and can be conveniently plugged and implemented in the flow cell. SARS-CoV-2 spike protein (10 nM, 20 nM, 35 nM, 45 nM, and 60 nM) and SARS-CoV-2 virus (5 × 10^3^ TCID_50_/mL, 9 × 10^3^ TCID_50_/mL, 2 × 10^4^ TCID_50_/mL, 4 × 10^4^ TCID_50_/mL and 6 × 10^4^ TCID_50_/mL) were used as target pathogens. Before testing, PBS was put into the flow cell to achieve the baseline signal of real-time detection. Then, the pathogens were slowly and continuously passed through the flow cell and incubated on the sensing probe for 20 min. Subsequently, the unbound target pathogens were washed off using PBS, a step that caused a slight downward shift in the signal. Next, another concentration of the target pathogen was tested. Repeat the above steps, recording the sensing signals for various analyte concentrations. The limit of detection is determined by the ratio of three times the standard deviation to the sensitivity (*S*). *S* is acquired as follows [32]:

(1) $$S=\frac{\lambda -\lambda_{0}}{C-0},$$

where *C* is the concentration of the tested target pathogen; *λ* is the response wavelength corresponding to *C*; and *λ*_0_ is the wavelength corresponding to blank samples without analytes. The limit of detection (*LOD*) is calculated as follows [33,34]:

(2) $$LOD=\frac{3\sigma }{S},$$

where *σ* is the standard deviation of the test response to the blank sample.

### 3.2. FO-SPR Sensing Probe Characterization

**Figure 3:**
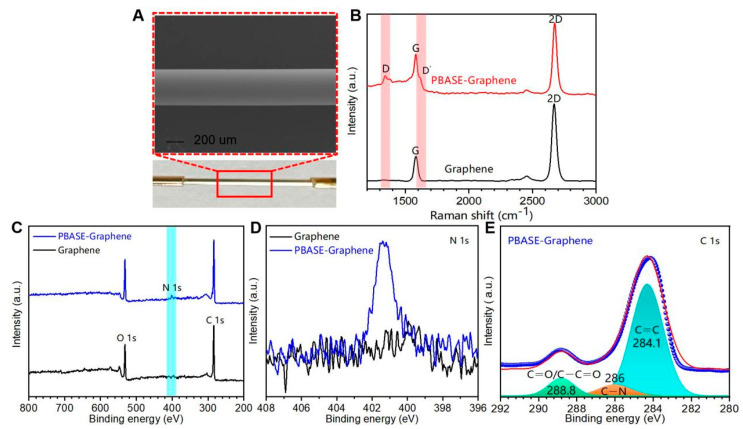
SEM, Raman spectra and XPS of pristine and modified FO-SPR sensing probe. (**A**) SEM image of modified FO-SPR sensing probe. (**B**) Raman spectra of pristine graphene (black) and PBASE-modified graphene (red). (**C**) XPS of pristine graphene (black) and PBASE-modified graphene (blue). (**D**) XPS N 1 s peaks of pristine graphene (black) and PBASE-modified graphene (blue). (**E**) Deconvolution of PBASE-modified graphene C 1 s peaks.

The monolayer graphene-coated FO-SPR sensing probe is shown in Figure 3. The PBSAE-graphene-modified FO-SPR sensing probe was analyzed by scanning electron microscopy (SEM), Raman spectra, and X-ray photoelectron spectroscopy (XPS). In Figure 3A, SEM shows that the surface of the graphene-coated sensing probe is uniform and pure. To confirm whether successfully modified PBASE on the graphene, the Raman spectrum and XPS are used to analyze the modified FO-SPR sensing surface (Figure 3B–E). The Raman spectra display the PBASE-modified graphene (red) and the pristine graphene (black), as shown in Figure 3B. In the pristine graphene Raman spectrum, there are two major G peaks (roughly at 1580.93 cm^−1^) and 2D peaks (roughly at 2671.32 cm^−1^). The G peak is attributed to the lattice vibrations of the sp^2^ carbon atoms and the second-order Raman scattering 2D peak [35,36]. After modification with PBASE, there are the D peak (roughly at 1340 cm^−1^) and D’ peak (roughly at 1615 cm^−1^). They are caused by the resonance modes of the pyrene group in PBASE interacting with the extended phonon modes of graphene [37,38]. In addition, the intensity ratio of graphene characteristic 2D and G peaks (*I*_2D_/*I*_G_) decreased from 3.08 (pristine graphene) to 1.24 after PBASE modification. The results indicate the presence of the doping effect on graphene [39]. At the same time, the ratio of *I*_2D_/*I*_G_ in Raman spectra confirms the monolayer structure of graphene [40]. The 2D peak is shifted to a higher frequency, which is attributed to the doping of graphene by the aromatic molecule PBASE through π–π stacking and tight binding to graphene [41,42,43]. Thus, the Raman spectra ensure the presence of PBASE on the FO-SPR probe.

XPS spectroscopy was used to analyze further the presence of PBASE on the graphene-coated FO-SPR sensing surface. Figure 3C shows the XPS spectra of PBASE-modified (blue) and pristine (black) graphene at the C 1 s, O 1 s, and N 1 s peaks. Since the only nitrogen source comes from the PBASE molecule, in Figure 3D, the distinct N1s peak (blue) confirms that PBASE is successfully loaded on the FO-SPR sensing region. In Figure 3E, the C 1 s peaks can be deconvoluted into three sub-peaks at around 284.1 eV, 286 eV, and 288.8 eV, which is attributed to the C 1 s orbital of C=C, C–N, C=O, and C–C=O, respectively [44]. The peak occurring at 284.1 eV is associated with the C=C aromatic rings of the sp^2^ graphene [45]. The C–N peak appears for the same reason as the N 1 s peak. The C=O/C–C=O is due to the residue of PMMA from the graphene-transferring process [31]. The results provide conclusive evidence that the loading of PBASE-graphene onto the FO-SPR sensing probe was flawless.

### 3.3. Experimental Verification of FO-SPR Sensing Device Performance

#### 3.3.1. Real-Time Detection of SARS-CoV-2 Spike S1 Protein

**Figure 4:**
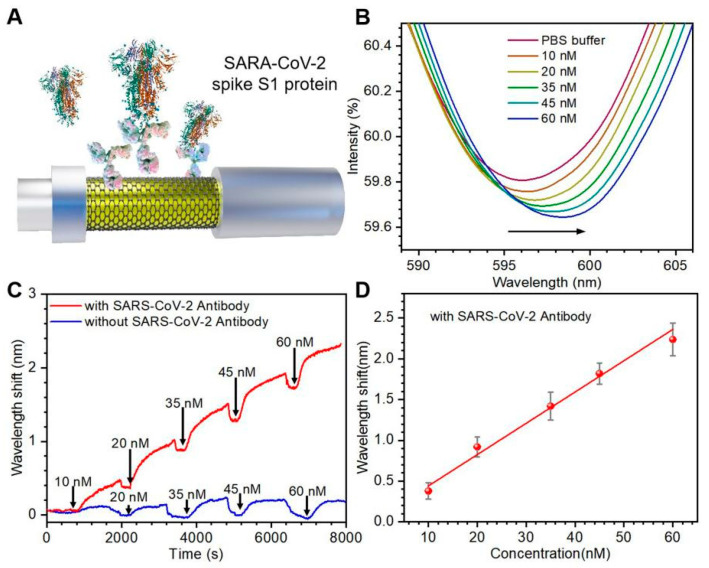
Testing SARS-CoV-2 spike S1 protein by portable FO-SPR device. (**A**) Schematic diagram of the antibody functionalized sensing probe. (**B**) SPR spectra of antibody functionalized sensing probes exposed to different concentrations of spike protein (10–60 nM). The arrow indicates the direction of the SPR signal offset. (**C**) Real-time wavelength shift curves with specific antibodies (red) and without specific antibodies (blue). (**D**) Linear fit of the maximum wavelength shift with spike protein concentrations. The error bars were determined by the standard deviation of three tests.

To evaluate the performance of the portable FO-SPR device, the SARS-CoV-2 spike protein was detected as the target pathogen. Figure 4A shows a schematic diagram of the antibody-functionalized FO-SPR sensing probe for detecting the SARS-CoV-2 spike protein. PBASE serves as a linker between graphene and antibody. The aromatic pyrenyl group of PBASE binds to the graphene through π–π interaction. Then, the SARS-CoV-2 spike S1 antibody binds with the PBASE through an amidation reaction [46]. In the sensing process, the SARS-CoV-2 spike protein was detected by sensing probes.

Figure 4B shows the SPR spectra curve of different SARS-CoV-2 spike protein concentrations. As the concentration of spike protein increases, the SPR resonance peak drifts toward the long-wave direction. It indicates that more spike protein was bound to the surface of the sensing probe. Figure 4C shows the wavelength real-time shift curve of the SPR spectrum acquired by the self-made program. The FO-SPR sensing probe functionalized with specific antibodies (red) has a significant signal response. By contrast, the sensing probe without functionalized specific antibody shows little noticeable signal responses (blue), and only a small amount of noise fluctuates slightly around the baseline. The experimental results indicate that the sensing probes can effectively prevent non-specific adsorption. In order to confirm whether the wavelength shifts of the SPR spectra can accurately respond to the change of spike protein concentration, a linear fit between the target pathogen concentration C and the maximum intensity shift (λ_max_) can be made as shown in Figure 4D. The fitted linear curve is as follows: λ_max_ = 0.04 × C + 0.06 (Correlation coefficient R^2^ = 0.988). The LOD is as low as 2.5 nM for spike protein in the linear range of 10–60 nM. The proposed FO-SPR device indicates a good linear relationship, making it suitable for precise analysis and detection of the target pathogen.

To showcase graphene modification can enhance the sensitivity, the FO-SPR sensing probe was coated with graphene and traditional 11-MUA, respectively. Figure S3A shows the wavelength shift of the probe exposed to the spike protein solution. The graphene-coated sensing probe has a higher wavelength shift than the traditional probe. In the linear range (Figure S3B), the LOD of the traditional FO-SPR sensing probe is more than 3-fold lower than that of the graphene-coated probe. The improved sensitivity is caused by the large specific surface area of graphene, which is beneficial for immobilizing more specific antibodies on the sensing surface, enabling more target pathogens to be captured.

**Figure 5:**
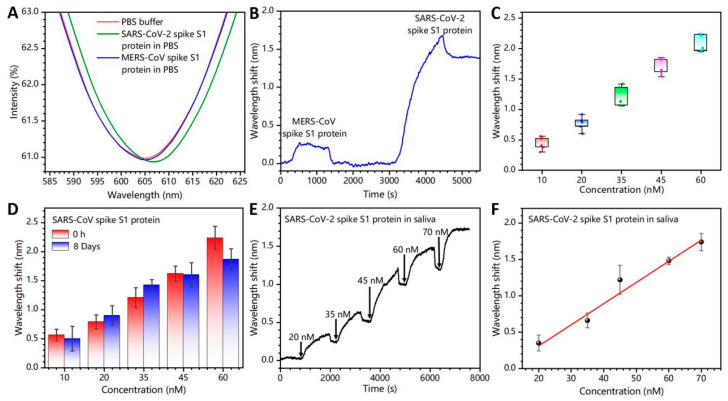
Selectivity testing (**A**) SPR spectra and (**B**) real-time response curves for SARS-CoV-2 spike S1 protein and MERS-CoV spike protein. (**C**) Repeatability test (the error bars were determined by the standard deviation of five tests). (**D**) Long-term stability tests for FO-SPR sensing probes stored in 4 °C PBS for 0 and 8 days. (**E**) Real-time response curves for testing of SARS-CoV-2 spike protein in artificial saliva. (**F**) Linear fit of the maximum wavelength shift with spike protein concentration. The error bars were determined by the standard deviation of three tests.

For verification of the selectivity, the sensing probes were exposed to 35 nM of MERS-CoV spike protein (control group) and SARS-CoV spike protein (experimental group) sequentially (Figure 5A,B). The SPR spectrum and the real-time signal response curve showed almost no wavelength shift when exposed to the MERS-CoV spike protein. This is ascribed to the absence of interaction between the functionalized sensing probes and the MERS-CoV spike protein. However, the SARS-CoV-2 spike antibody can bind and block the binding epitopes (e.g., receptor binding domains (RBDs) and other related domains on the SARS-CoV-2 spike protein), demonstrating the remarkable response of the SPR spectrum to the SARS-CoV-2 spike antibody. Thus, the portable device shows good selectivity for the target pathogens compared with the control group.

Repeatability and long-term stability of bio-functionalized FO-SPR devices are also essential parameters. We evaluated the repeatability by testing the SARS-CoV-2 spike protein under identical experimental conditions at various times (Figure 5C). Five measurements were performed on each sample, and the relative standard deviation (*RSD*) was calculated to obtain the device-to-device repeatability, which was denoted as “100%–*RSD*”. The *RSD* is expressed as follows:

(3) $$RSD=\frac{SD}{\bar{X}}\times 100\%,$$

where $\bar{X}$ is the average value of wavelength variation for each experimental group; and *SD* is the standard deviation. Table S1 shows the repeatability of the device from 77.2–93.2%. Then, the sensing probes were stored in PBS solution at 4 °C for 8 days to verify its long-term stability. Figure 5D shows that the sensing probe still maintains a good detection ability for target pathogens after long-term storage. The results indicate the portable device has stable detection capability and it has promising prospects in clinical application for medical diagnostics.

To evaluate the reliability and accuracy of the FO-SPR device in more realistic situations, the SARS-CoV-2 spike protein was tested in a complex artificial saliva environment. The results demonstrate that the FO-SPR device could successfully detect SARS-CoV-2 spike proteins in saliva (Figure 5E). The LOD was 6.1 nM. Subsequently, a fitted linear curve with R^2^ of 0.987 was obtained (Figure 5F). It indicates that the device could avoid interference from other biological components and may be used for highly infectious pathogens content determination and screening.

#### 3.3.2. Real-Time Detection of Inactivated SARS-CoV-2 Virus

**Figure 6:**
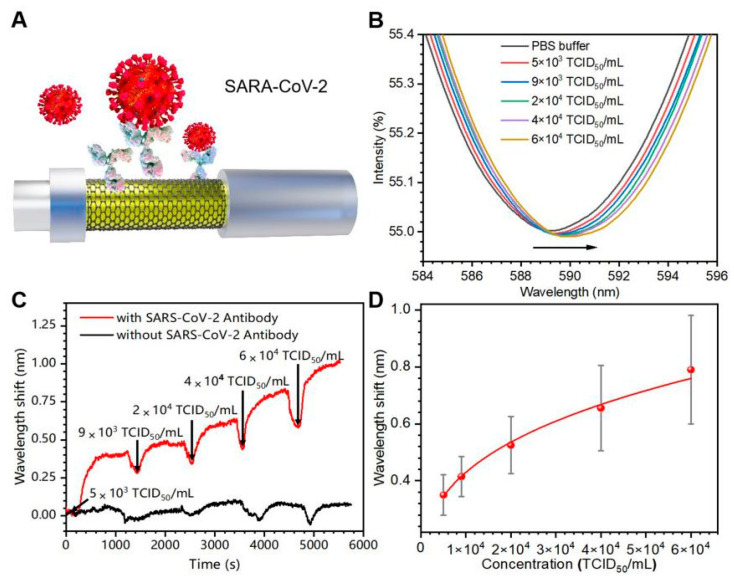
Testing of SARS-CoV-2 inactivated virus in PBS. (**A**) Schematic diagram of the antibody functionalized FO-SPR sensing probe for detecting SARS-CoV-2 virus. (**B**) SPR spectra for different SARS-CoV-2 virus concentrations (5 × 10^3^ TCID_50_/mL to 6 × 10^4^ TCID_50_/mL). The arrow indicates the direction of the SPR signal offset. (**C**) Real-time wavelength shift curves with specific antibodies (red) and without specific antibodies (black). (**D**) Linear fit of the wavelength shifts to different SARS-CoV-2 concentrations. Error bars were determined from the standard deviation of three tests.

Finally, the inactivated SARS-CoV-2 virus was tested via FO-SPR device (Figure 6A). The change of SPR spectra with the concentrations change of SARS-CoV-2 virus (5 × 10^3^ TCID_50_/mL to 6 × 10^4^ TCID_50_/mL) are shown in Figure 6B. The sensing probes without specific antibodies were used as negative controls. Figure 6C shows the wavelength shift curve of the sensing probe with specific antibodies (red), exhibiting a significant response compared with the negative control (black). A fitted curve with R^2^ of 0.994 was obtained; λ_max_ = 0.02 × C^0.32^ (Figure 6D). LOD was 2.2 × 10^3^ TCID_50_/mL. This demonstrates that the FO-SPR device could detect SARS-CoV-2 in real time with high selectivity and has a bright potential application in POCT for highly infectious pathogens.

## 4. Conclusions

In conclusion, a portable POCT device has been developed for highly infectious pathogen detection. The device integrates an FO-SPR sensing component, a signal acquisition and processing system, and an embedded power supply unit, all controlled by a self-made program. It offers rapid data visualization for users’ timely inspection results. The compact design enhances the convenience of pathogen detection. Moreover, the modification of monolayer graphene contributes to improving the detection sensitivity. The device achieves direct detection of target pathogens without labels within 20 min. For SARS-CoV-2 spike S1 protein detection, LOD is 2.5 nM in PBS and 6.1 nM in artificial saliva. The LOD of the SARS-CoV-2 virus is 2.2 × 10^3^ TCID_50_/mL. The LOD of the FO-SPR device is similar to other reported SPR biosensors (Table S2). The device exhibits high selectivity for target pathogens, as demonstrated via cross-reactivity experiments with SARS-CoV-2 spike protein and MERS-CoV spike protein. The portable POCT device also presents satisfactory long-term stability (8 days) and repeatability (77.2–93.2%). The above results indicate that the successful development of the portable FO-SPR device provides a reliable technological approach for rapid on-site detection of highly infectious pathogens. This research has great potential to improve the response capacity of infectious disease emergencies and ensure public health security.

## Data Availability

Data are contained within the article and Supplementary Materials.

## Supplementary Materials

The following supporting information can be downloaded at https://www.mdpi.com/article/10.3390/bios13121029/s1: Figure S1: Scheme of the graphene-coated FO-SPR sensing probe; Figure S2: Scheme of the bio-functionalized FO-SPR sensing probe; Figure S3: Testing SARS-CoV-2 spike S1 protein by portable FO-SPR device; Table S1: RSD for repeatability analysis; Table S2: Comparison of SPR methods for biosensing analysis [47,48,49,50,51,52,53,54].
