# Supplementary material for: A Point-of-Care Testing Device Utilizing Graphene-Enhanced Fiber Optic SPR Sensor for Real-Time Detection of Infectious Pathogens
Source: Biosensors (Basel). 2023 Dec 14;13(12):1029. doi: 10.3390/bios13121029 (PMC10741924; doi:10.3390/bios13121029)
Supplement: Supplementary file 1 [file biosensors-13-01029-s001.zip › biosensors-2743620-supplementary.pdf]

Supplementary Material

A point-of-care testing device utilizing graphene-enhanced fiber optic SPR sensor for real-time detection of infectious pathogens

Shiyu Jiang [1,2], Siyu Qian [1,*], Shunning Zhu [1], Jinxin Lu [1], Yunxin Hu [1,2], Cheng Zhang [3], Yikai Geng [1], Xuefeng Chen [1], Ying Guo [1], Zhaoliang Chen [3], Jie Pu [3], Zhendong Guo [3] and Shengchun Liu [1,*]

[1] Heilongjiang Provincial Key Laboratory of Metamaterials Physics and Device, Heilongjiang University, Harbin, 150080, China
[2] School of Electronic Engineering, Heilongjiang University, Harbin, 150080, China
[3] Changchun Veterinary Research Institute, Chinese Academy of Agricultural Sciences, Changchun, 130122, China
* Correspondence: liushengchun@hlju.edu.cn (S. C. Liu), qiansiyu@hlju.edu.cn (S. Y. Qian).

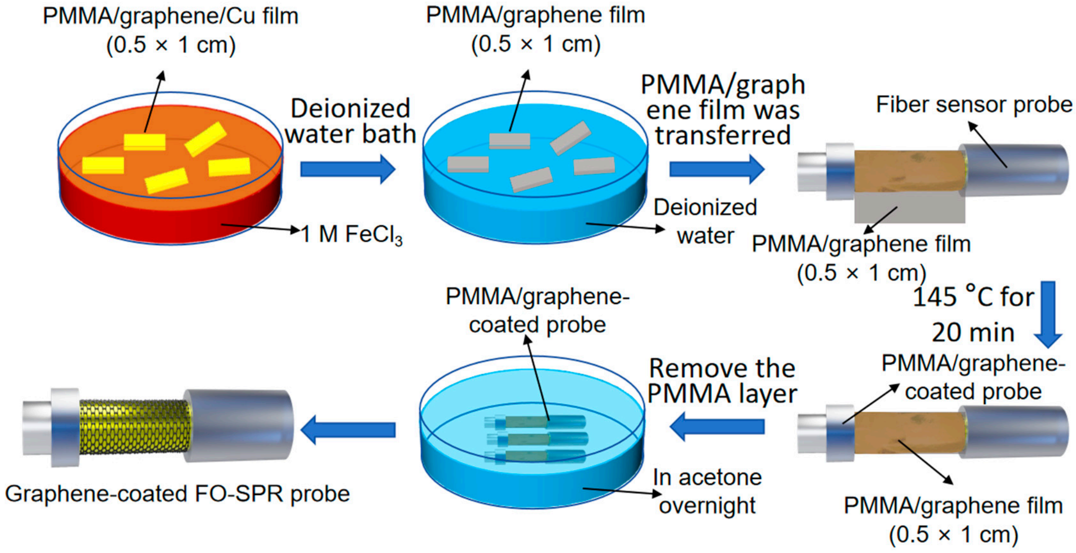

Figure S1. Scheme of the graphene-coated FO-SPR sensing probe.

11-MUA-modified FO-SPR sensing probes.

The FO-SPR sensing probe with bare gold was immersed in 1 mM 11-MUA in ethanol at room temperature overnight and alternately rinsed with ethanol and deionized water. Then, to activate the carboxyl group, the FO-SPR probe was placed in an aqueous 0.5 M NHS/EDC solution for 20 min and rinsed with deionized water. The sensing probe was immersed in 250 μg/mL SARS-CoV-2 spike S1 antibody in PBS for 45 min. Finally, the probe was put in 1 mg/mL BSA for 1 h.

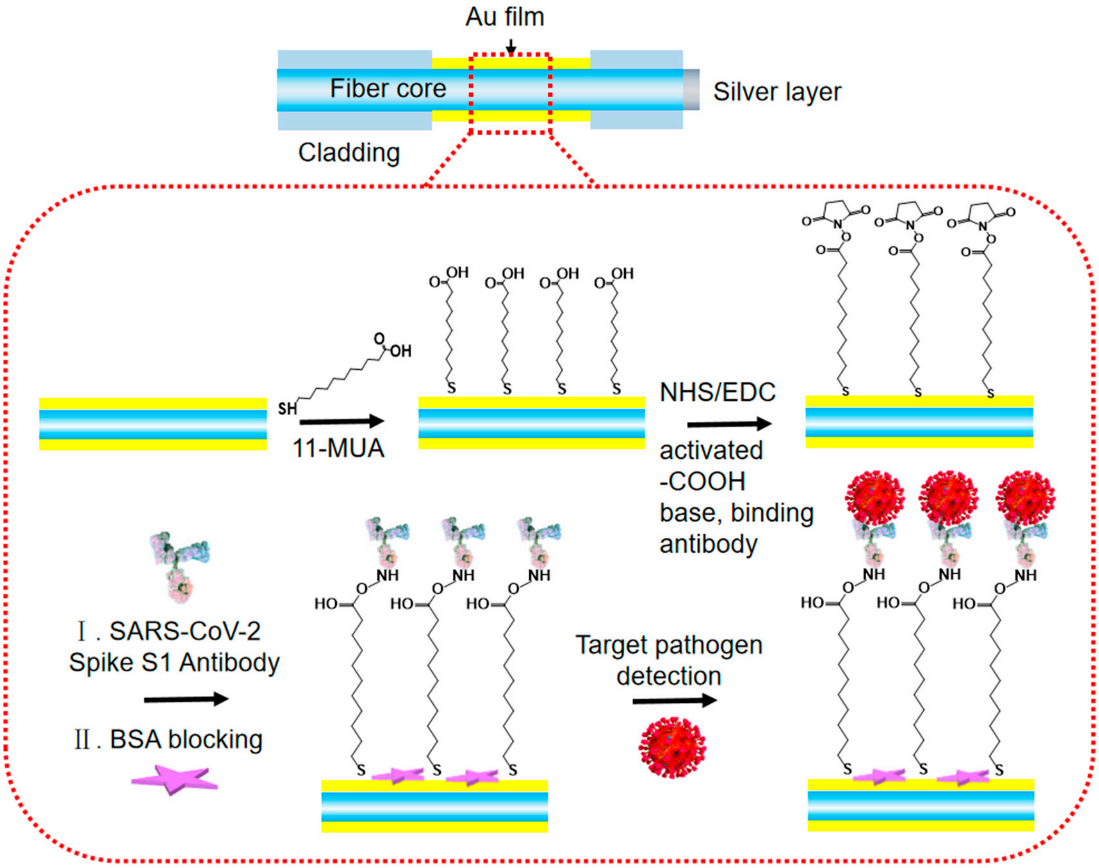

Figure S2. Scheme of the bio-functionalized FO-SPR sensing probe.

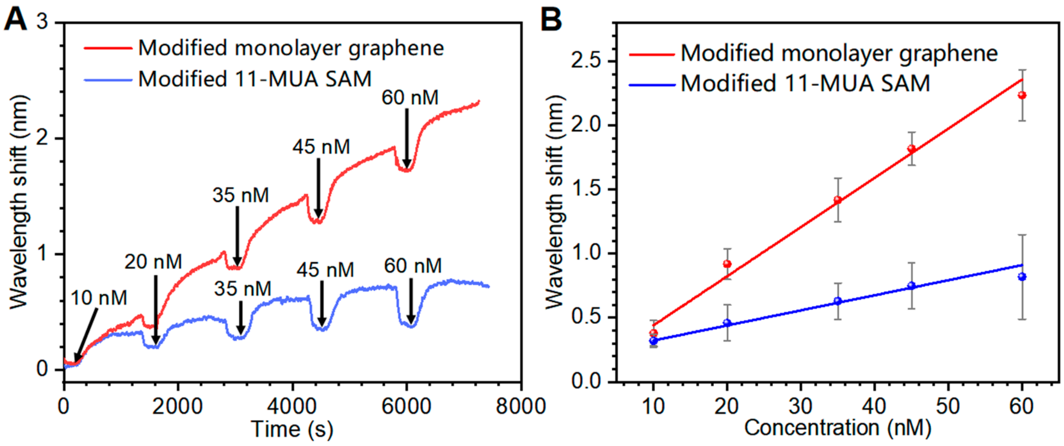

Figure S3. Testing SARS-CoV-2 spike S1 protein via portable FO-SPR device. (A) The graphene-coated FO-SPR sensing probe wavelength shift curve (red) and the conventional 11-MUA-modified FO-SPR sensing probe wavelength shift curve (blue). (B) The graphene-coated FO-SPR sensing probe (red) and the conventional 11-MUA-modified FO-SPR sensing probe (blue) were linearly fitted for different protein concentrations and wavelength shifts. Error bars were determined by the standard deviation of three tests.

Table S1. RSD for repeatability analysis.

SARS-CoV-2 spike S1 protein concentration	Relative standard deviation (RSD)	Repeatability (100%-RSD)
10 nM	22.8	77.2
20 nM	15.5	84.5
35 nM	12.2	87.8
45 nM	8.6	91.4
60 nM	6.8	93.2

Table S2. Comparison of SPR methods for biosensing analysis.

Method	Type of receptor	Target analyte	Linear range	Limit of detection	Ref
SPR	Escherichia coli	Phage T4	0 PFU/mL - 2×10^8 PFU/mL	1×10^7 PFU/mL	[1]
SPR	polyclonal antibody	Salmonella enterica	-	10^5 CFU/mL	[2]
LSPR	Casein	Trypsin	0.1 μg/mL - 100 μg/mL	4.3 nM	[3]
SPR	Hb antibody	Hb	5 μg/mL - 250 μg/mL	0.078 μM	[4]
SPR	Antibody	HIV-1 p24 capsid protein	-	4.1 ± 0.5 nM	[5]
FO-SPR	SPA	IgG	30 μg/mL - 100 μg/mL	0.5 μg/mL	[6]
FO-SPR	DNA-aptamer	SARS-CoV-2 spike protein	25 nM - 1 μM	37 nM	[7]
FO-SPR	Anti-ADM monoclonal antibody	ADM	-	0.25 μg/mL	[8]
FO-SPR POCT	SARS-CoV-2 spike S1 protein antibody	SARS-CoV-2 spike S1 protein<br>SARS-CoV-2 virus	10 nM - 60 nM<br>5 × 10^3 $TCID_{50}$/mL - 6 × 10^4 $TCID_{50}$/mL	2.5 nM<br>2.2 × 10^3 $TCID_{50}$/mL	This work

LSPR: Localized surface plasmon resonance; HIV: Human immunodeficiency virus; SPA: Staphylococcal protein A; Hb: hemoglobin; IgG: Human immunoglobulin G; ADM: Adalimumab.

References

[1] Xiao, C.Q.; Jiang, F.L.; Zhou, B.; Li, R.; Liu, Y. Immobilization of Escherichia coli for detection of phage T4 using surface plasmon resonance. Science China Chemistry 2012, 55, 1931-1939. https://doi.org/10.1007/s11426-012-4553-6.

[2] Makhneva, E.; Farka, Z.; Skládal, P.; Zajíčková, L. Cyclopropylamine plasma polymer surfaces for label-free SPR and QCM immunosensing of Salmonella. Sensors and Actuators B: Chemical 2018, 276, 447-455. https://doi.org/10.1016/j.snb.2018.08.055.

[3] Svärd, A.; Neilands, J.; Palm, E.; Svensäter, G.; Bengtsson, T.; Aili, D. Protein-Functionalized Gold Nanoparticles as Refractometric Nanoplasmonic Sensors for the Detection of Proteolytic Activity of Porphyromonas gingivalis. ACS Applied Nano Materials 2020, 3, 10, 9822-9830. https://doi.org/10.1021/acsanm.0c01899.

[4] Inci, F.; Saylan, Y.; Kojouri, A.M.; Ogut, M.G.; Denizli, A.; Demirci, U. A disposable microfluidic-integrated hand-held plasmonic platform for protein detection. Applied Materials Today 2020, 18, 100478. https://doi.org/10.1016/j.apmt.2019.100478.

[5] Sarcina, L.; Mangiatordi, G.F.; Torricelli, F.; Bollella, P.; Gounani, Z.; Österbacka, R.; Macchia, E.; Torsi, L. Surface Plasmon Resonance Assay for Label-Free and Selective Detection of HIV-1 p24 Protein. Biosensors 2021, 11, 6, 180. https://doi.org/10.3390/bios11060180.

[6] Wang, Q.; Jing, J.Y.; Wang, B.T. Highly Sensitive SPR Biosensor Based on Graphene Oxide and Staphylococcal Protein A Co-Modified TFBG for Human IgG Detection. IEEE Transactions on Instrumentation and Measurement 2019, 68, 9, 3350-3357. https://doi.org/10.1109/TIM.2018.2875961.

[7] Cennamo, N.; Pasquardini, L.; Arcadio, F.; Lunelli, L.; Vanzetti, L.; Carafa, V.; Altucci, L.; Zeni, L. SARS-CoV-2 spike protein detection through a plasmonic D-shaped plastic optical fiber aptasensor. Talanta 2021, 233, 122532. https://doi.org/10.1016/j.talanta.2021.122532.

[8] Qu, J.H.; Ordutowski, H.; Tricht, C.V.; Verbruggen, R.; Gallardo, A.B.; Bulcaen, M.; Ciwinska, M.; Cisneros, C.C.; Devriese, C.; Guluzade, S.; Janssens, X.; Kornblum, S.; Lu, Y.H.; Marolt, N.; Nanjappan, C.; Rutten, E.; Vanhauwaert, E.; Geukens, N. Thomas, D.; Dosso, F.D.; Safdar, S.; Spasic, D.; Lammertyn, J. Point-of-care therapeutic drug monitoring of adalimumab by integrating a FO-SPR biosensor in a self-powered microfluidic cartridge. Biosensors and Bioelectronics 2022, 206, 114125. https://doi.org/10.1016/j.bios.2022.114125.
